# Resonant noise amplification in a predator-prey model with quasi-discrete generations

**DOI:** 10.1038/s41598-024-67098-3

**Published:** 2024-07-22

**Authors:** M. Giannakou, B. Waclaw

**Affiliations:** 1 School of Physics and Astronomy, University of Edinburgh, James Clerk Maxwell Building, Peter Guthrie Tait Road, Edinburgh, EH9 3FD UK; 2 Dioscuri Centre for Physics and Chemistry of Bacteria, Institute of Physical Chemistry PAS, Kasprzaka 44/52, 01-224 Warsaw, Poland; 3 Institut für Physik, Johannes Gutenberg-Universität Mainz, Staudingerweg 9, 55128 Mainz, Germany

**Keywords:** Biological physics, Statistical physics

## Abstract

Predator-prey models have been shown to exhibit resonance-like behaviour, in which random fluctuations in the number of organisms (demographic noise) are amplified when their frequency is close to the natural oscillatory frequency of the system. This behaviour has been traditionally studied in models with exponentially distributed replication and death times. Here we consider a biologically more realistic model, in which organisms replicate quasi-synchronously such that the distribution of replication times has a narrow maximum at some $T>0$ corresponding to the mean doubling time. We show that when the frequency of replication $f=1/T$ is tuned to the natural oscillatory frequency of the predator-prey model, the system exhibits oscillations that are much stronger than in the model with Poissonian (non-synchronous) replication and death. These oscillations lead to population instability and the extinction of one of the species much sooner than in the case of Poissonian replication. The effect can be explained by resonant amplification of coloured noise generated by quasi-synchronous replication events.

## Introduction

When a non-linear dynamical system capable of exhibiting damped oscillations is coupled to a source of random noise, it often begins to generate periodic oscillations (a quasi-cycle)^1^. This resonance-like behaviour is caused by the amplification of noise frequencies that are in tune with the natural oscillatory frequency of the system. Importantly, the noise does not have to be external but it can be intrinsic to the system and arise from its microscopic stochastic dynamics.

An important example is resonant amplification of demographic noise which has been found in stochastic models of biological populations^2–8^. However, all these models assume that reproduction is a Markov process: birth and death occur with certain (possibly state-dependent) rates. At any moment, the distribution of replication times is therefore exponential, with the maximum at time $t=0$. However, biological organisms do not replicate in this way: all known organisms require a certain minimum time to develop reproductive capability. Moreover, many organisms reproduce in quasi-discrete generations such that the time between consecutive replication events has a narrow distribution that peaks around some characteristic time *T* called the generation time, or doubling time. For example, for the bacterium *E. coli*, *T* ranges between 20 min and a few hours and the coefficient of variation of the doubling time is 0.1–0.3, depending on growth conditions^9,10^. This leads to significant correlations between reproduction times of related individuals. Modelling this process for a single species has a long history^11–17^.

The quasi-synchronous nature of replication suggest an interesting possibility: if a predator-prey system has a tendency to oscillate at a frequency similar to the inverse of the doubling time, synchronisation of the two oscillations may lead to a substantial enhancement of resonant amplification of demographic noise.

In this work, we investigate this scenario in a simple predator-prey model originally proposed in Ref.^2^. The model assumes two biological species interacting in a way that leads to damped predator-prey cycles in the limit of infinitely large populations. In the original model, demographic noise due to stochastic replication of organisms led to persistent oscillations of small but non-zero amplitude and a Lorenz-like power spectrum. Here we show that when replication is no longer Poissonian but occurs in quasi-discrete generations, these oscillations increase dramatically in amplitude and can be as high as 50% of the total population size even when the number of organisms is very large (millions or more). This in turn destabilizes the coexistence of the two species and can lead to one of the species going extinct even for large population sizes that would make extinction unlikely in the case of Poissonian replication.

## Two-species model with quasi-synchronous replication

Our model is an extension of the Newman-McKane model^2^. We consider a well-mixed population of two types of organisms, A and, B that replicate in quasi-discrete generations with the same mean doubling time, and die randomly with rates dependent on the current state of the system. We shall call these organisms “cells” as if they were single-celled microorganisms, although the model is agnostic to the exact nature of these organisms.

We assume that type A’s death rate decreases in the presence of B proportionally to the concentration of B. This could be due to type B producing an essential chemical compound necessary for A to survive. Type B, on the other hand, is killed by A (e.g., A releases a toxin that kills B) with rate proportional to the abundance of A. The death rate of B also increases due to crowding. Similar interactions, including prey-predator type interactions between two microbial species, have been demonstrated in microbial populations^18–22^.

More specifically, let $N_A,N_B$ be the number of cells of each type. Each cell has an internal state variable $\tau$ assigned at birth from a certain distribution $R(\tau )$, the same for both species. We shall call this variable a “timer”. The timer counts down from the assigned time interval; when it reaches zero, the cell produces an offspring and both cells are assigned new, randomly selected values of $\tau$ from $R(\tau )$. We will consider two choices for the distribution $R(\tau )$: (i) exponential (the Poisson model) with mean time to division *T*, (ii) uniform on $(T(1-w),T(1+w))$ where $w\ll 1$ controls the degree of correlation of replication times. See SI Section S1 for a more detailed discussion of these distributions.

Cells die with per-capita rates $d_A=p_2 - p_1 N_B/K$ for type A, and $d_B=p_4 N_A/K - p_3(1-N_B/K)$ for type B. Here *K* plays a role similar to the carrying capacity in population dynamics models and sets the scale for the number of cells in the system: $N_A,N_B\sim K$ on average. We have used the same symbols for the parameters $p_1,p_2,p_3,p_4$ as in Ref.^2^, however their microscopic interpretation is slightly different. We will come back to this when we discuss the steady-state solution of the model.

**Figure 1:**
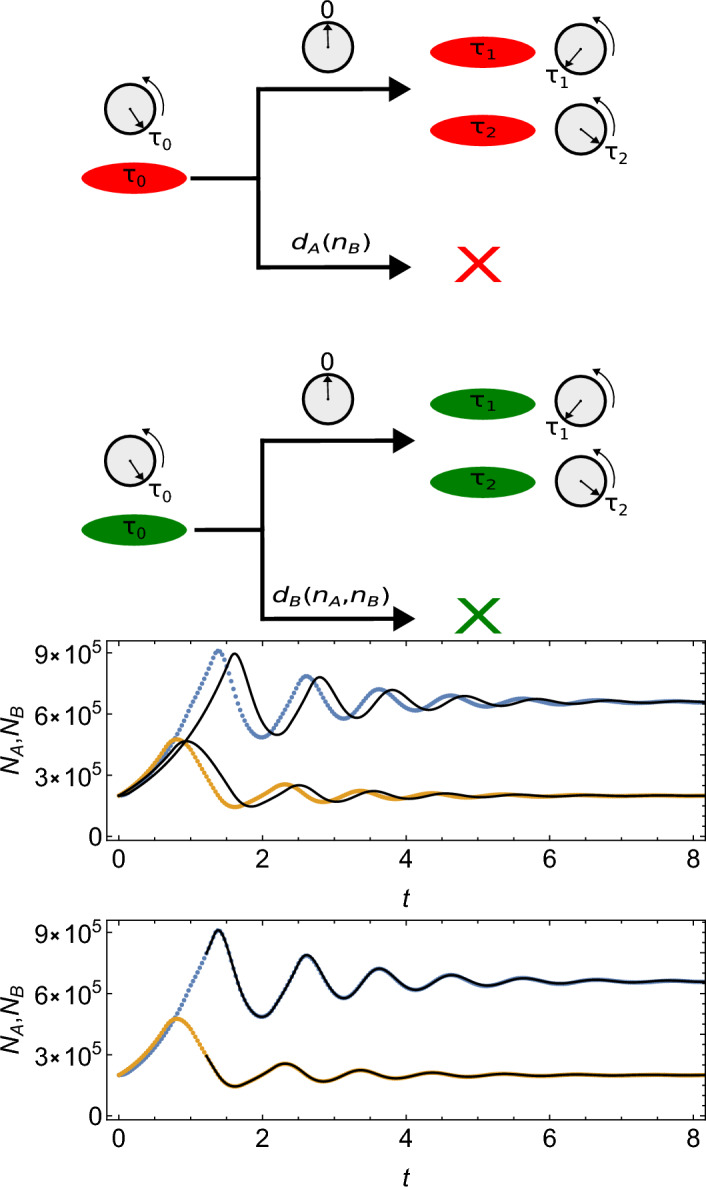
Top: Graphical representation of all processes in the model. Red/green represent cells of type A and B, respectively. Each cell has an internal clock variable $\tau$ counting down to zero (division) with the same rate for all cells. Upon division, two daughter cells are assigned random clock variables $\tau _1$ and $\tau _2$ from the probability distribution $R(\tau )$. Death occurs randomly with rates $d_A, d_B$ that depend on the number of cells of each type. Middle and bottom: Damped oscillations in the stochastic asynchronous model (points: blue for $N_A$, yellow for $N_B$) and its deterministic counterpart (black lines), for $\{p_1,p_2,p_3,p_4,b\}=\{30,7,7,10,1\}$. Middle: the deterministic solution differs from the stochastic simulation for the uniform initial distribution of the timer variable. Both models assume the same initial condition $x_A(0)=0.2,x_B(0)=0.2$. Bottom: the agreement is very good when we compare the models after the stochastic model has reached a quasi-steady state timer distribution. The deterministic model uses the values of $N_A,N_B=\{ 797423, 293636\}$ from the stochastic simulation for $t_0=1.2207$ as its initial condition.

Figure 1, top, schematically represents all interactions in the model. We note that while death is an inhomogeneous Poisson process occurring with state-dependent rates as in Ref.^2^, replication is not since it depends on the internal state $\tau$ of each cell. The model is thus non-Markovian.

### Mean-field approximation shows the effect of synchronicity on short-term population dynamics

We first study the behaviour of the Poisson model in the infinite-population size limit ($K\rightarrow \infty$) by neglecting fluctuations in the number of cells. We define $x_A=N_A/K, x_B=N_B/K$ as the new state variables. The dynamics of the model can be approximated by two differential equations:

1 $$\begin{aligned} \frac{dx_A}{dt}= & {} x_A \left( b - \max [ p_2 - p_1 x_B, 0] \right) , \end{aligned}$$

2 $$\begin{aligned} \frac{dx_B}{dt}= & {} x_B \left( b - \max [p_4 x_A - p_3(1-x_B),0] \right) , \end{aligned}$$

where $b=\ln (2)/T$. In the above, we have used average rates of all processes and assumed that all higher moments factorize into products of $x_A,x_B$. The $\max [\dots ]$ function ensures that the death terms contribute only if the corresponding rates are positive. Equations (1, 2) thus give the standard mean-field approximation of the stochastic model.

The non-zero steady-state solution of the model, with both species present, reads:

3 $$\begin{aligned} x_A^*= & {} \frac{(p_1-p_2)p_3+b(p_1+p_3)}{p_1p_4}, \end{aligned}$$

4 $$\begin{aligned} x_B^*= & {} \frac{p_2-b}{p_1}. \end{aligned}$$

To investigate the stability of this solution, we Taylor-expand equations (1-2) around the steady-state solution. This leads to the Jacobian matrix with the following eigenvalues:

5 $$\begin{aligned} \lambda _\pm =\frac{b p_3 - p_2 p_3 \pm \sqrt{\Delta }}{2 p_1}, \end{aligned}$$

where $\Delta =(b-p_2) \left( b (2 p_1 +p_3)^2 - p_3 \left( -4 p_1^2 + 4 p_1 p_2 + \right. \right.$ $\left. \left. + p_2 p_3\right) \right)$. In the regime that we are interested in ($x_A^*>0, x_B^*>0$), the eigenvalues have a negative real part, meaning that the steady-state solution is stable to a small perturbation. However, their imaginary part is generally non-zero, hence the system will exhibit damped oscillations while relaxing towards the steady state. This is illustrated in Fig. 1, which shows plots of the deterministic solution for $\{p_1,p_2,p_3,p_4,b\}=\{30,7,7,10,1\}$, for a short time (a few oscillations), starting from $x_A(0)=0.2,x_B(0)=0.2$. The same figure shows the results of a numerical simulation of the stochastic Poisson model with $K=10^6$. Note the small discrepancy between both models (Fig. 1, middle). This is due to the timer variable of all cells being initialized with uniformly distributed random numbers in the stochastic simulation. This initial distribution is quite different from the quasi-steady state distribution obtained after a few cycles, which has been implicitly assumed when deriving Eqs. (1, 2) from the microscopic rules given in the beginning of Section 2. When we solve the deterministic model starting from $N_A,N_B$ taken at a later time point during the simulation of the stochastic model, the discrepancy vanishes (Fig. 1, bottom).

### Automated scanning of the parameter space selects parameters in the oscillatory regime

The deterministic model has five parameters: $p_1,p_2,p_3,p_4,b$. We can put $b=1$; this fixes the time scale. The remaining four parameters determine the frequency of small-amplitude oscillations, the damping coefficient, and steady-state occupations. We shall now discuss how we select these parameters so that the model exhibits under-damped oscillations; this is required for the resonant amplification of noise. We use Eq. (5) together with Eqs. (3–4) to find a region in the parameter space $\{p_1,p_2,p_3,p_4\}$ and $b=1$ of the deterministic model that corresponds to damped oscillations of frequency $f_0=\textrm{Im}(\lambda )/(2\pi )\in (0.98,1.02)$, damping coefficient $|\textrm{Re}(\lambda )|<0.5$, and steady-state abundances $0.5\pm 0.1$. We do this via Monte-Carlo sampling of the parameter space. We then use the selected values as starting points for a root finding algorithm to find ${p_1,p_2,p_3,p_4}$ such that the frequency is exactly $f_0=1$, steady-state occupations $x_A=x_B=0.5$, and the damping coefficient assumes one of three values: 0.5 (fast damping), 0.2 (slow damping) and 0.1 (minimal damping).

This procedure has produced three sets of parameters: $S_{0.5}=\{39.73, 20.86, 2., 4.\}$, $S_{0.2}=\{56.45, 29.23, 0.8, 2.8\}$, and $S_{0.1}=\{65.81, 33.91, 0.4, 2.4\}$. We shall use these parameters in the full, stochastic model.

## Results of computer simulations of the model

Standard kinetic Monte Carlo algorithms such as the original Gillespie algorithm^23^ cannot be used because the model is generally not Markovian. We have therefore simulated the model using an algorithm with a fixed-size time step $dt=1/512$. The clock variable advances deterministically, whereas the numbers of organisms to be killed are generated by drawing binomially-distributed random numbers, with $N_A,N_B$ being the number of trials, and success probabilities equal to the corresponding death rate multiplied by *dt*.

### Synchronous replication significantly increases the magnitude of oscillations

**Figure 2:**
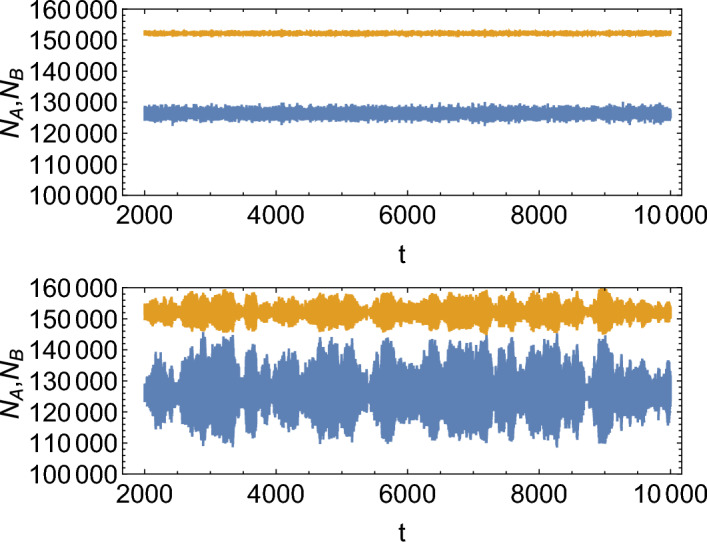
Example time series $N_A(t),N_B(t)$ in the Poisson (top) and quasi-synchronous (bottom) models, for $S_{0.5}$, $K=300000, w=0.02$, and the initial condition $N_A(0)=0.2K, N_B(0)=0.2K$. Individual oscillations cannot be seen due to the length of the time window shown here; the window contains a few thousand oscillations such as those visible in Fig. 1.

Figure 2 shows examples of time series obtained for the Poissonian version of the model, and for the quasi-synchronous model with a narrow distribution of doubling times. In both cases the natural oscillatory frequency of the system is $f_0=1$ and the average doubling time is $T=1$.

**Figure 3:**
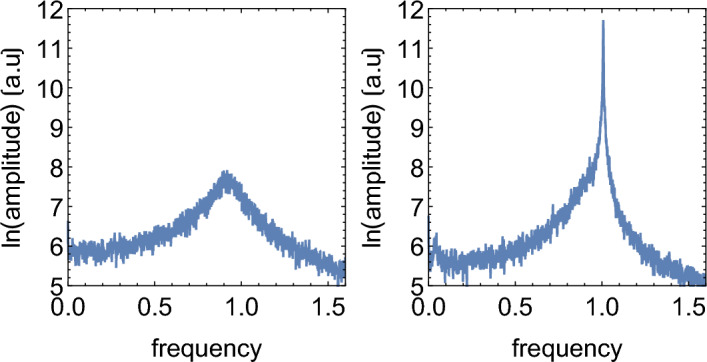
Fourier spectrum of $N_A(t)$ in the Poisson (left) and non-Poisson (right) models, for $K=300000,w=0.02$, and the remaining parameters are $S_{0.5}$. Moving average has been applied to smooth out the spectra.

The quasi-synchronous model exhibits much larger oscillations than the Poissonian model. Accordingly, the Fourier spectrum of the quasi-synchronous model has a much more pronounced peak at the fundamental frequency $f_0=1$ (Fig. 3). The observed increase in the amplitude of oscillations occurs only when the doubling frequency is close to the natural oscillation frequency. Figure  4, top, shows that when $T\ne 1/f_0$ the amplitude is significantly reduced; this resonance-like behaviour is not present in the Poisson model (black line in Fig. 4, top). Interestingly, the maximum amplitude is observed at a slightly lower $b=0.5$ than expected ($b=\ln 2=0.69$ which corresponds to $T=1$). The resonance peak is also quite broad. This is a non-linear effect; for large amplitudes as observed here, the resonant frequency is slightly lower than $f_0=1$.

**Figure 5:**
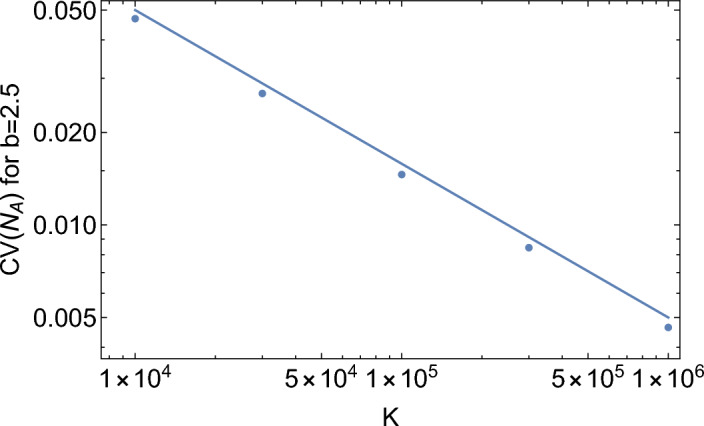
Coefficient of variation of $N_A(t)$ for the quasi-synchronous model as a function of *K*, calculated at $b=2.5$ (away from the resonance peak). Solid line represents the scaling $\sim 1/\sqrt{K}$ expected for the Poisson version of the model.

The resonance peak becomes sharper with increasing carrying capacity *K* (Fig. 4, middle). The amplitude of oscillations near the resonance peak is independent of *K* for a wide range of *K*. This is very different to the scaling $\sim 1/\sqrt{K}$ observed in the Poisson case and also the scaling of the coefficient of variation (CV, the standard deviation divided by the mean) in the quasi-synchronous model far away from the peak (Fig. 5). The amplitude of oscillations in the quasi-synchronous model is more than 10% of the steady-state population abundance for $K=10^6$, whereas in the Poisson model with identical parameters it is less than 1%.

Figure 4, bottom, shows that the height of the resonance peak decreases with increasing *w*. For $w=0.2$, the peak is barely noticeable. On the other hand, all values of $w\le 0.1$ produce a visible peak.

**﻿Figure 4:**
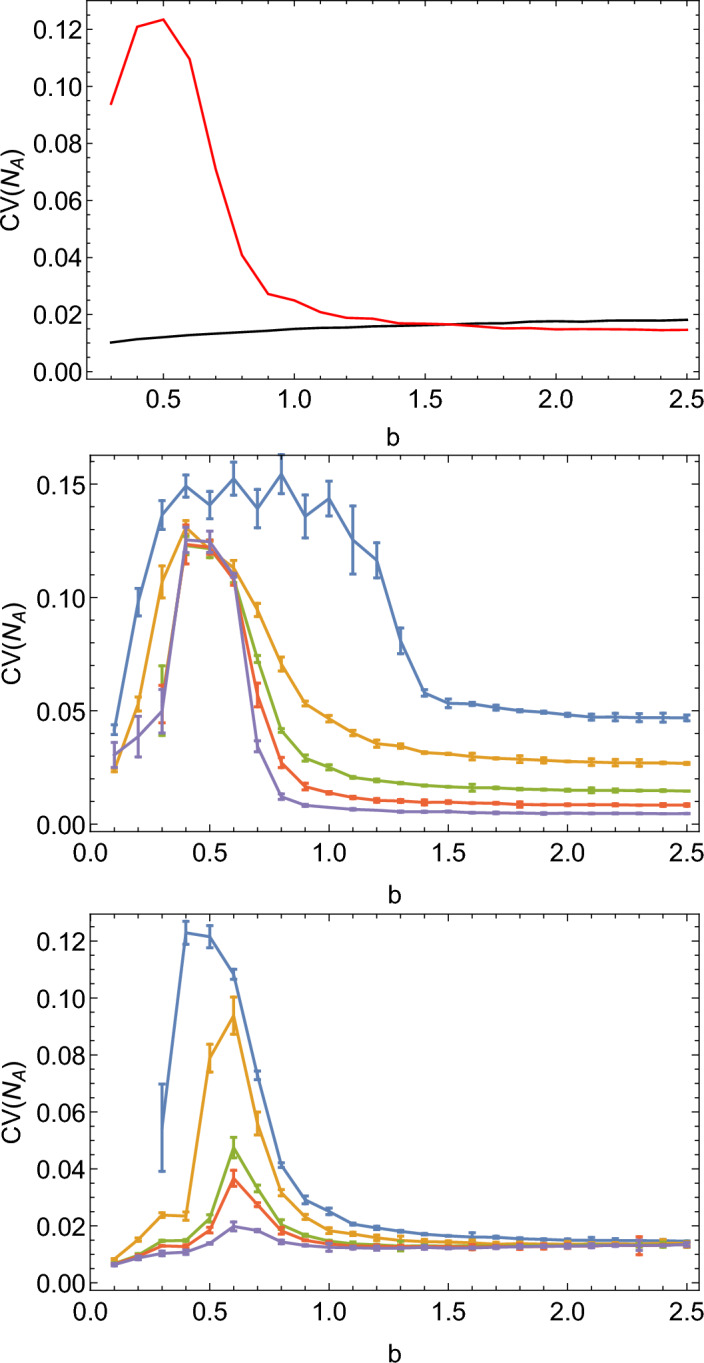
Top: Coefficient of variation (CV) of $N_A(t)$ for the Poisson (black) and quasi-synchronous (red) models, as a function of *b*. CV is a convenient measure of the amplitude of oscillations. A resonance peak can be seen at $b\approx 0.5$. Middle: CV of $N_A(t)$ for the quasi-synchronous model with $w=0.02$ and different $K=10^4,3\times 10^4,10^5, 3\times 10^5, 10^6$ (blue, yellow, green, red, violet). Bottom: CV versus *b* for different widths $w=0.02,0.04,0.08,0.1,0.2$ of the doubling time distribution (colours from blue to violet) and $K=10^5$. In all cases, parameters = $S_{0.5}$.

These results suggest that quasi-synchronous replication leads to a significant enhancement of demographic noise in the quasi-synchronous model. To qualitatively understand this, let us first revisit what happens in the Poisson version of the model^2^. In that model, demographic noise has a flat spectrum and contains a broad range of frequencies (white noise). Frequencies close to the frequency at which the system exhibits damped oscillations are amplified; this leads to quasi-periodic oscillations with the amplitude $\sim \sqrt{K}$. However, since the average abundances $N_A,N_B$ increase proportionally to *K*, the relative magnitude of oscillations decreases as $\sim 1/\sqrt{K}$ with the increasing population size. Noise-induced oscillations are therefore significant only for relatively small systems $K\ll 10^6$. In contrast, here we observe large, persistent oscillations even for $K=10^6$. As we shall see, this can be explained by demographic noise being concentrated in a narrow range of frequencies in the non-Poisson model. This effect becomes the stronger, the narrower (smaller *w*) the replication time distribution $R(\tau )$ is which explains why the resonance peak decreases in magnitude as *w* increases in Fig. 4.

**Figure 6:**
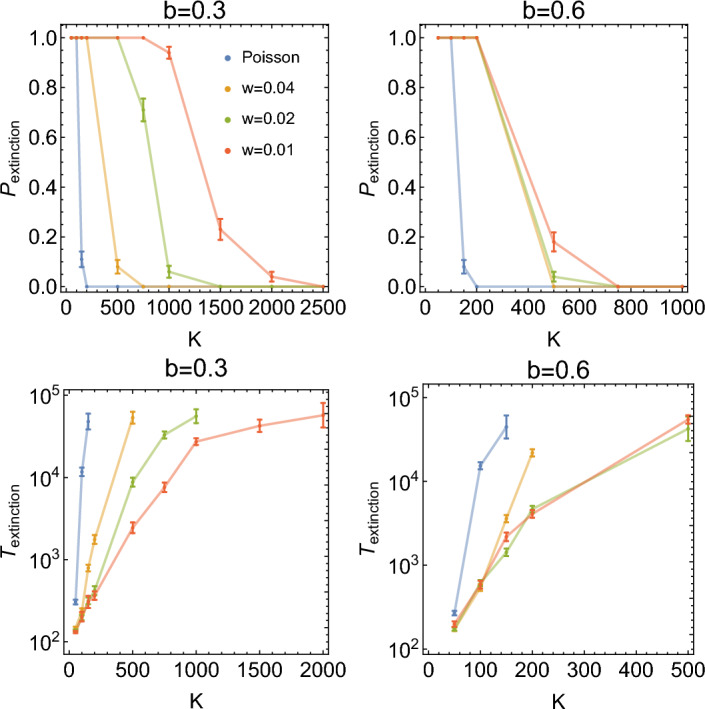
Top: extinction probability during the time interval $T_{\textrm{max}}=10^5$ versus *K*, for different *w*. Left: $b=0.3$, right: $b=0.6$. Bottom: average time to extinction versus *K*, for the same parameters as the top row of plots. Due to the finite simulation time ($T_{\textrm{max}}=10^5$), the average extinction time appears to be skewed towards lower values. Other parameters are as in $S_{0.5}$.

### Enhanced demographic noise leads to increased extinction probability

A population of stochastically reproducing and dying organisms may be prone to extinction^24,25^. This is especially significant for small populations with large fluctuations in the number of organisms. In Markovian models, in which the fluctuations are typically of the order of $\sim \sqrt{K}$, the mean time to extinction is $T_{\textrm{extinction}}\sim \exp (a K)$, where the coefficient *a* depends on the microscopic details of the model^24,26,27^.

Since quasi-synchronous replication significantly enhances the amplitude of oscillations of $N_A,N_B$, we expect that it will also decrease $T_{\textrm{extinction}}$ or, equivalently, increase the probability of the population going extinct during a fixed period of time. However, in our two-species model, we do not expect both species to go extinct at the same time; rather, we will call an extinction event if any of the two species goes extinct. Such an event does not necessary cause the population to die out as the other species may still live for a very long time, but it destroys species coexistence^28^.

Our expectations are confirmed in Fig. 6, top, in which we plot the extinction probability as a function of carrying capacity *K*, for different *w* (different levels of replication synchrony), during a fixed time interval $T_{\textrm{max}}=10^5$. The capacity *K* at which the extinction probability is non-zero increases significantly with increasing synchronisation (decreasing *w*). This means that synchronous replication makes extinction more likely even for relatively large population sizes, for which Poissonian replication is extremely unlikely to cause extinction. We also note that for our specific set of parameters $S_{0.5}$, it is almost always (i.e., in all simulation runs performed) species A that becomes extinct, whereas species B continues to live.

Interestingly, the effect of increased synchronicity (decreased *w*) is more pronounced further from resonance ($b=0.3$) than close to it ($b=0.6$), for which the extinction probability curves are similar for different $w\ll 1$. The same behaviour can be seen when we plot the time to extinction versus *K*, for the same two values of *b* (Fig. 6, bottom).

**Figure 7:**
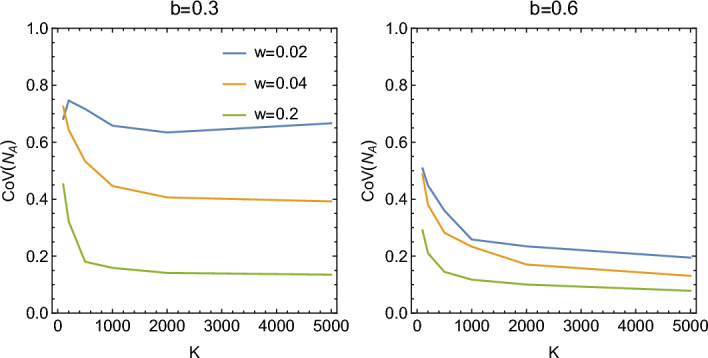
Coefficient of variation of $N_A$ versus *K*, for different *w*. Left: $b=0.3$, right: $b=0.6$.

The lack of sensitivity to the level of synchrony for *b* close to resonance could be explained by our observation from the previous section that for small *K* the amplitude of oscillations increases non-linearly with decreasing *w*; even a moderate amount of synchronicity is enough to bring about large oscillations of $N_A(t)$, which can lead to extinction. To check how large these oscillations are, we plotted the coefficient of variation of $N_A(t)$ versus *K*, for $b=0.3$ and $b=0.6$ (Fig. 7). We indeed see that the curves for different *w* are closer to each other for $b=0.6$ (close to resonance) than for $b=0.3$.

## Mathematical theory of quasi-synchronous replication

To gain a better understanding of how demographic noise is enhanced by quasi-synchronous replication, we will develop a mathematical theory describing various aspects of the model.

### A single-species model offers insight into the nature of noise

We start by considering a simpler one-species model, in which replication is non-Poissonian with mean doubling time $\ln (2)/b$, whereas death is a Poisson process with rate *bN*/*K*, where *N* is the total number of cells, *K* is the carrying capacity, and *b* is the replication rate. In the large-*K* limit, the average abundance $x=N/K$ evolves according to the logistic equation,

6 $$\begin{aligned} dx/dt = bx(1-x). \end{aligned}$$

The steady-state occupation is $x^*=1$. In the stochastic model ($K<\infty$), the number of cells is thus expected to fluctuate around the mean value $N^*\cong K$.

**Figure 8:**
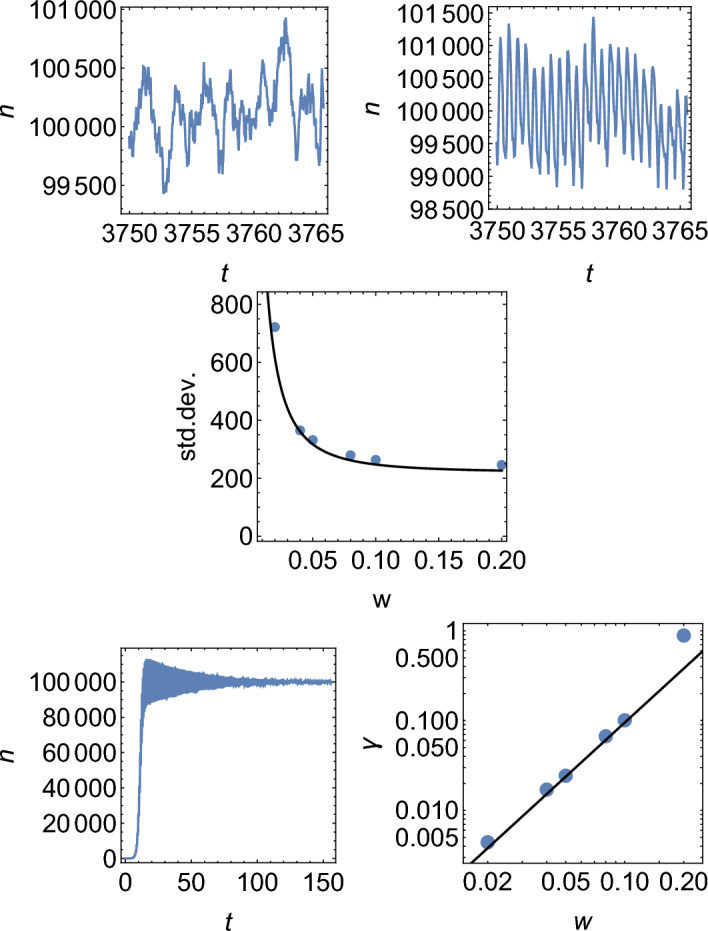
Fluctuations in the single-species model. Top: *N*(*t*) for $K=100000$: Poisson model (left), and quasi-synchronous model with $w=0.02$ (right). Middle: standard deviation of fluctuations for different *w*. Points = simulation, line = equation (12) with $\gamma = 9.5w^2$. Bottom left: exponential decay of oscillation in the single-species model for $K=100000$ and $w=0.04$. Bottom right: the exponential decay rate $\gamma$ as a function of *w* (points). Black line is the theoretical prediction $\gamma \approx 9.5w^2$; no fitting has been performed here.

Figure 8, top, shows examples of *N*(*t*) for the model with Poisson and non-Poisson replication ($w=0.02$), for $K=10^5$. The quasi-synchronous model exhibits more regular oscillations. The standard deviation of *N*(*t*) is very similar to the Poisson model for $w>0.05$ but rapidly increases for smaller *w* (Fig. 8, middle).

To obtain the relationship between *w* and the amplitude of oscillations, we begin by considering the behaviour of a large population of cells in which the cells can be assigned to groups depending on the phase $\phi$ of their cell cycle. The phase is not the same as the timer variable; instead, it should be interpreted as the difference between the timer variable and some arbitrary chosen reference timer. Let $n(\phi )$ be the number density of cells with phase $\phi$. Let us further assume that, if all cells were synchronised (all $\phi$ being equal), the total number of cells would be described by a certain periodic function *f*(*t*). This function (besides a different amplitude) also describes demographic fluctuations in a group of cells that have the same phase $\phi$. The total number of cells in the population is therefore

7 $$\begin{aligned} N(t)=\int _{-\infty }^{\infty } n(\phi )f(t-\phi )d\phi , \end{aligned}$$

which is the convolution of *f* and *n*. The Fourier spectrum of *N* is

8 $$\begin{aligned} {\mathcal {F}}[N](\omega )={\mathcal {F}}[f](\omega ) \,{\mathcal {F}}[n](\omega ). \end{aligned}$$

Suppose $n(\phi )$ is the Gaussian distribution with variance $\sigma ^2$. We have

9 $$\begin{aligned} {\mathcal {F}}[N](\omega )={\mathcal {F}}[f](\omega ) e^{-(1/2)\sigma ^2 \omega ^2}. \end{aligned}$$

If *f* is periodic with angular frequency $\omega _0$, then the lowest-frequency Fourier mode of *N* at $\omega =\omega _0$ will be reduced in comparison to *f* by $e^{-(1/2)\sigma ^2 \omega ^2}$ due to the spread of the phases. All higher modes will be damped even more; we will neglect them for now. For $\omega _0=2\pi /\ln 2$ assumed in our single-species model for $b=1$ (doubling time $=\ln 2$) and $\sigma ^2\ll 1$, the reduction factor is $e^{[2\pi ^2/(\ln 2)^2]\sigma ^2}\approx e^{-41.1\sigma ^2}$.

The finite width of the distribution of doubling times causes the distribution *n* to broaden every generation, so that $\sigma ^2=\sigma _0^2 (t/ \ln (2))$, where $\sigma _0^2=(w^2/3)(\ln 2)^2$ is the variance of the uniform distribution of doubling times used in the simulations. This corresponds to the variance of the phase distribution increasing by the variance of the doubling time distribution every generation. This will then lead to oscillations in *N*(*t*) (caused by quasi-synchronous replication) to decay exponentially with the rate $\gamma =[2\pi ^2/(\ln 2)^2](\ln 2)(w^2/3) = [2\pi ^2/(3\ln 2)]w^2 \approx 9.5w^2$. Figure 8, bottom, shows that the predicted rate is in very good agreement with the decay rate observed in numerical simulations.

If we now consider death as the source of perturbations, demographic noise at any given time point will have two contributions: random death events happening now, and death-induced perturbations to $n(\phi )$ from previous cycles. The latter are amplified by a factor of two every cycle (cell replication) but are also damped with rate $\gamma (w)$. We therefore expect the average spectrum $\left<|{\tilde{N}}|^2(f)\right>$ to have a *w*-independent contribution due to white noise caused by death, and a Lorentzian contribution due to the amplified noise from previous cycles:

10 $$\begin{aligned} \left<|{\tilde{N}}|^2(f)\right>_{\textrm{Lorentzian}} \sim \frac{1}{(2\pi f-\omega _0)^2+\gamma ^2(w)}, \end{aligned}$$

where *f* is the frequency and $\omega _0=2\pi /\ln 2$ is the replication angular frequency defined earlier. The Lorentzian part comes about from the lowest-Fourier mode approximation to the evolution equation for $\Delta N(t)=N(t)-\left<N(t)\right>$: $\Delta N''(t)=-\omega _0^2 \Delta N(t) - \gamma \Delta N(t)$ plus periodic forcing with frequency *f*. The relative contribution of these two terms will depend on *w*. By virtue of Parsival theorem, the variance $\left<(N(t)-\left<N(t)\right>)^2\right>=\left<|\Delta N(t)|^2\right>$ can be written as:

11 $$\begin{aligned} \left<|\Delta N(t)|^2\right>\sim & {} K \left( 1 + c_0 \int \left<|{\tilde{N}}|^2(f)\right>_{\textrm{Lorentzian}} df\right) \nonumber \\\sim & {} K(1+c_0/(2\gamma )), \end{aligned}$$

with some constant $c_0$. Since the perturbation decay rate $\gamma$ decreases with *w*, for sufficiently synchronous replication (small *w*), the term $\sim 1/\gamma$ will dominate and demographic noise in the system will be significantly amplified compared to the Poissonian case.

The above result can be derived more formally, see SI Sec. S2. In particular, we obtain that

12 $$\begin{aligned} \left<|\Delta N|^2\right> \cong K (0.48+0.0125/\gamma ), \end{aligned}$$

and the demographic noise spectrum can be approximated by

13 $$\begin{aligned} \left<|{\tilde{N}}|^2(f)\right> \cong K\left( \frac{0.962}{1+(2\pi f)^2} + \frac{0.0231336}{(2\pi f-\frac{2\pi }{\ln 2})^2+\gamma ^2} \right) . \end{aligned}$$

Figures 8 and S2 show that equations (12) and (13) agree well with the numerically obtained variance and spectrum, for a broad range of *w* values.

### The behaviour of two-species model can be derived from the single-species model

We now go back to the two-species model. We shall assume that near the steady state the dynamics can be described by linearized equations

14 $$\begin{aligned} dy_A/dt= & {} a_{AB}y_B + \eta _A, \end{aligned}$$

15 $$\begin{aligned} dy_B/dt= & {} a_{BA}y_A + a_{BB} y_B + \eta _B, \end{aligned}$$

where $y_A=x_A-x_A^*, y_B=x_B-x_B^*$ ($x_A^*,x_B^*$ are steady-state concentrations), and $a_{AB},a_{BA},a_{BB}$ are given by the following expressions

16 $$\begin{aligned} a_{AB}= & {} \frac{p_3 (b-p_2)+p_1(b+p_3)}{p_4}, \end{aligned}$$

17 $$\begin{aligned} a_{BB}= & {} \frac{p_3 (b-p_2)}{p_1}, \end{aligned}$$

18 $$\begin{aligned} a_{BA}= & {} \frac{p_4 (b-p_2)}{p_1}. \end{aligned}$$

Here $\eta _A,\eta _B$ represent noise (not necessarily white noise) due to replication and death of both species.

Fourier-transforming Eqs. (14, 15) leads to the following expression for the spectrum of $y_A$:

19 $$\begin{aligned}{} & {} \left<|{\tilde{y}}_A(\omega )|^2\right> \nonumber \\{} & {} \quad = \left[ (a_{BB}^2+\omega ^2) \left<|\eta _A|^2\right> -2 a_{AB} a_{BB} \left<|\eta _A\eta _B|^2\right> \right. \nonumber \\{} & {} \qquad +\left. a_{AB}^2\left<|\eta _B|^2\right> \right] / \left[ a_{AB}^2 a_{BA}^2+2 a_{AB} a_{BA} \omega ^2 \right. \nonumber \\{} & {} \qquad \left. +a_{BB}^2 \omega ^2+\omega ^4\right] . \end{aligned}$$

**Figure 9:**
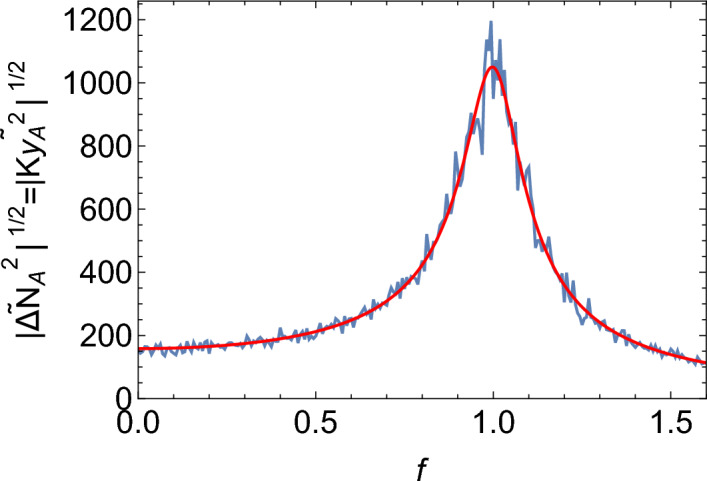
Plot of the spectrum of $y_A$ versus the frequency $f=\omega /(2\pi )$, for the Poisson model. Blue = simulation with $K=10^5,b=1$, and the remaining parameters as in $S_{0.5}$. Red = analytic expression (19) with $\left<|\eta _A|^2\right>=\left<|\eta _B|^2\right>=bK$, and $\left<|\eta _{AB}|^2\right>=0$.

The formula for the spectrum of $y_B$ is very similar (not shown). Figure 9 shows that Eq. (19) works well for the Poisson case (asynchronous replication), for which we assume $\left<|\eta _A|^2\right>=\left<|\eta _B|^2\right>=(2b)(K/2)=bK$ (recall that *K*/2 is the average number of organisms for our choice of the parameters, and the factor 2*b* is due to both birth and death contributing equally near the steady state), and $\left<|\eta _{AB}|^2\right>=0$.

**Figure 10:**
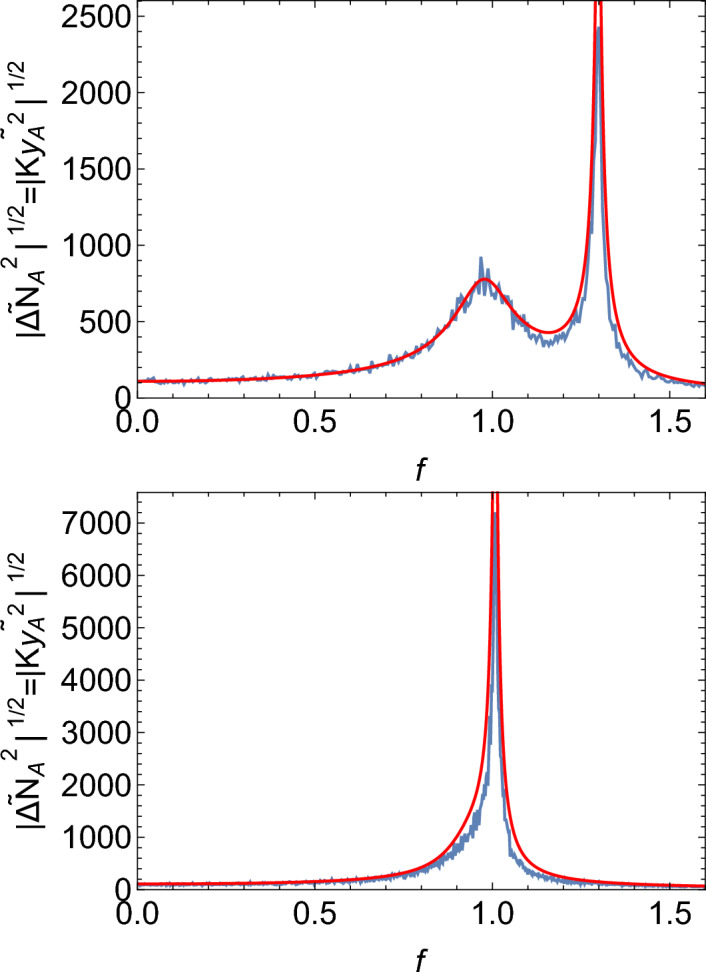
Plot of the spectrum of $y_A$ versus frequency $f=\omega /(2\pi )$, for the non-Poisson model with the replication frequency slightly detuned ($b=0.9$, top) and in resonance ($b=0.7$, bottom). Blue = simulations for $K=10^5, w=0.08$, and the remaining parameters as in $S_{0.5}$. Red = analytic expression 19 with the noise terms from Eq. (S63), and $\gamma =6.579w^2$ (here $T=1$).

Interestingly, Eq. (19) also works for the non-Poissonian case, with an appropriate choise of the coloured noise $\eta _A,\eta _B$, based on the single-species calculation presented in the previous section and in SI Section S2 E. The calculation is shown in SI Section S3 A. Figure 10 demonstrates this for different replication frequencies (controlled by *b*). If the replication frequency is slightly detuned from the natural frequency of the system (Fig. 10, top), two peaks are visible in the spectrum: a sharp peak coming from the quasi-synchronous birth events, and a much wider but lower peak corresponding to white noise-induced oscillations at the natural frequency $f\approx 1$.

Figure 10, bottom, shows that when $b=0.7$ is tuned in to the resonant frequency of the system, only one peak is visible, with a slight broadening towards lower frequencies.

**Figure 11:**
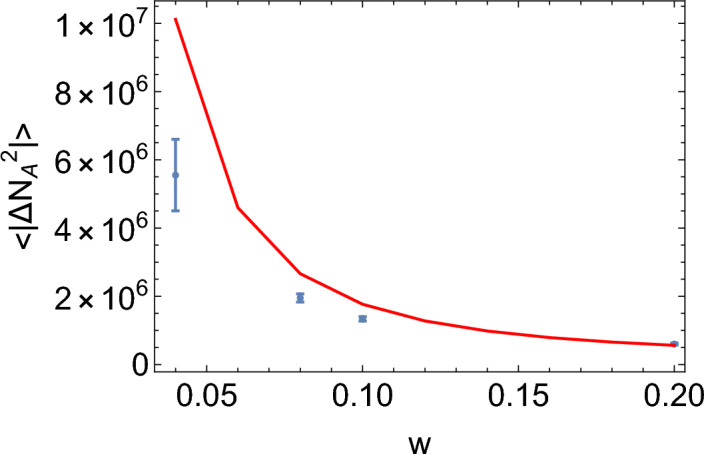
Plot of $\left<|\Delta N_A(t)|^2\right>$ versus *w*, for the non-Poisson model with replication frequency $b=0.7$. Blue = simulation results for $K=10^5$, and the remaining parameters as in $S_{0.5}$. Red = analytic expression from Eq. (S64).

We can also calculate the amplitude of fluctuations (SI Section S3 B). Figure 11 shows the plot of $\left<|\Delta N_A(t)|^2\right>$ obtained in this way, compared to the simulation results. We notice that the analytic formula correctly reproduces the trend but the theoretically predicted values are generally larger than the ones from the simulation. Note that we used equation (S64), which is the same as the formula for the Poisson case^2^, but with coloured noise given by Eq. (S63) instead of white noise. The fact that this approach works means that oscillations in the system with quasi-synchronous replication can be understood as being caused by resonant amplification of coloured, non-Poissonian noise.

To get some qualitative insight into the role of $\gamma$, we consider the case $\gamma \ll 1$. For $T=1, b=\ln 2$, we can expand the formula under the integral in Eq. (S64) around $\omega =2\pi$, which enables us to carry out the integral analytically. We obtain

20 $$\begin{aligned}{} & {} \left<|\Delta N_A(t)|^2\right> \cong \left[ KD_2\pi ^2 (a_{BB}^2 + a_{AB}^2 + 4\pi ^2) (\ln 2)^2\right] \nonumber \\{} & {} \quad / \left[ \gamma (a_{AB}^2 a_{BA}^2 + 4 (2 a_{AB} a_{BA} + a_{BB}^2)\pi ^2 + 16 \pi ^4) \times (4 \pi ^2 + (\ln 2)^2)\right] . \end{aligned}$$

We see that, since $\gamma \sim w^2$, the variance of $\Delta N_A$ increases as $1/w^2$ as reproduction becomes progressively more synchronous for $w\rightarrow 0$. This is similar to the effect of a long delay in reaction kinetics^29^. The relationship $\left<|\Delta N_A(t)|^2\right> \propto K/\gamma$ can be interpreted as an effective reduction in the number of replicating entities; cells originating from a common ancestor replicate quasi-synchronously when their sub-population is much less than $1/\gamma$. The system thus consists of $K\gamma \ll K$ of such groups of cells, which increases demographic noise by a factor $1/\sqrt{\gamma }$, and the variance of *N* by $1/\gamma$.

We can also ask how small *w* must be to significantly increase demographic noise. In SI Section S3 C we show that the contribution to the variance of $N_A$ from synchronous replication becomes comparable to the contribution from stochastic death for $\gamma <2/\sqrt{c}$, or when $w< 0.55 c^{-1/4}$, with a positive $c=-a_{AB} a_{BA} a_{BB}^2 - a_{BB}^4/4$. As the expression is rather insensitive to the value of *c*, we can conclude that deviations from the Poisson, asynchronous replication should already be visible even for relatively large *w* (low synchronicity).

## Conclusion

We have revisited the stochastic two-species model of the predator-prey type from Ref.^2^, which exhibits oscillations for a wide range of parameters of the model. We have modified the model so that both species replicate quasi-synchronously, with doubling times drawn from a narrow distribution. We have shown that coloured demographic noise generated by this process leads to much stronger oscillations than the Poisson process of replication assumed in earlier works. Coloured noise has been shown to affect population dynamics in single-species models^30^; here, we not only derive its spectrum from the underlying microscopic dynamics, but also show how it affects more complex models.

We have also seen that quasi-synchronous replication increases the extinction probability of one of the species due to stochastic fluctuations in the number of organisms. Extinction in multi-species stochastic models have been investigated before^28^. Our result shows that the effect of demographic noise in such systems is even more dramatic in the case of synchronous replication.

Our results may be relevant for real biological populations of microorganisms which often replicate in quasi-discrete generations. Since, as we show, even moderate amount of synchonisation can visibly increase fluctuations in the number of organisms and the probability of extinction, synchronous replication should be taken into account when creating models of microbial population dynamics. We also expect to see a very similar behaviour in other models that exhibit quasi-cycles^28,31–35^.

The phenomenon of coloured noise amplification may be further enhanced in situations in which oscillations in the population abundance become synchronized with reproductive cycles. We leave this interesting problem for future studies.

### Supplementary Information

Supplementary Information 1.

## Data Availability

All data and code presented have been deposited in a GitHub repository and can be accessed via: https://github.com/Dioscuri-Centre/SynchonousReplication. Documentation is also found in the link which explains further the data and code.
