# Supplementary material for: Resonant noise amplification in a predator-prey model with quasi-discrete generations
Source: Sci Rep. 2024 Jul 22;14:16783. doi: 10.1038/s41598-024-67098-3 (PMC11263699; doi:10.1038/s41598-024-67098-3)
Supplement: Supplementary file 1 — Supplementary Information 1. [file 41598_2024_67098_MOESM1_ESM.pdf]

Supplementary Information
Resonant noise amplification in a predator-prey model with quasi-discrete generations

M. Giannakou[1,3], B. Waclaw[1,2]

[1] *School of Physics and Astronomy, University of Edinburgh, James Clerk Maxwell Building, Peter Guthrie Tait Road, Edinburgh, EH9 3FD, United Kingdom*

[2] *Dioscuri Centre for Physics and Chemistry of Bacteria, Institute of Physical Chemistry PAS, Kasprzaka 44/52, 01-224 Warsaw, Poland*

[3] *Institut für Physik, Johannes Gutenberg-Universität Mainz, Staudingerweg 9, 55128 Mainz, Germany*

S1. CHOICE OF THE DISTRIBUTION OF REPLICATION TIMES

Let us briefly discuss some possible choices for the distribution $R(\tau)$ of replication times. The case $R(\tau) = \delta(\tau - T)$ corresponds to all cells reproducing in perfect synchrony; the generation time is T. The exponential distribution $R(\tau) = (1/T)\exp(-\tau/T)$ represents the Poisson case: cells reproduce with rate $1/T$ per capita and the mean time to replication is T. In this case the model is Markovian and its behaviour is expected to be the same as the original model from Ref. [2]. Finally, $R(\tau)$ can be concentrated around $\tau = T$ but have a non-zero width. This represents quasi-synchronous replication: all descendants of a given cell initially replicate in quasi-discrete generations, with progressive loss of synchronization over time.

In this manuscript, we compare the behaviour of the model for two distributions $R(\tau)$: (i) exponential (the Poisson model) with mean time to division T and (ii) uniform on $(T(1-w), T(1+w))$, where $w \ll 1$ controls the degree of correlation of replication times. The choice of a uniform distribution simplifies calculations. However, as demonstrated in Sec. IV A, our results depend mainly on the variance of $R(\tau)$ and not its exact form. Therefore, we expect the results to be qualitatively similar for other single-peaked and sufficiently narrow distributions.

S2. ANALYTICAL DERIVATIONS FOR THE SINGLE-SPECIES MODEL

We note that non-Markovian models involving delayed reactions and power spectra have been considered before [36]. The approach presented below differs from that earlier work because, in contrast to Ref. [36], we do not assume that replication involves an additional Poisson step. While it might be technically possible to recast our model into the form required for the method of Ref. [36], the resulting calculation would have a similar complexity as the one presented in the paper, while it would not provide the additional insight of our current approach.

We shall start by writing down the equation for the number density of cells $n(\tau, t)$ at time t with the timer variable τ, for the time being neglecting stochastic noise:

$$\partial_t n(\tau,t) = \partial_\tau n(\tau,t) - \frac{\ln 2}{T} n(\tau,t) \int_0^\infty \frac{n(\tau',t)}{K} d\tau' + 2R(\tau)n(0,t). \quad \text{(S1)}$$

The first term corresponds to the timer counting backward. The second term represents death with rate proportional to the total size divided by K. The factor $(\ln 2)/T$ is required to have the correct behaviour in the limit of perfectly synchronous replication - we shall see this later. The third term represent replication that occurs when the timer reaches $\tau = 0$ and is the product of the density of cells $n(0, t)$ in that state and $R(\tau)$, the probability density function for the timer being reset to τ. We assume $R(\tau)$ to be normalized:

$$\int_0^\infty R(\tau) d\tau = 1, \quad \text{(S2)}$$

and that $R(\tau)$ is concentrated around $\tau = T$ as in numerical simulations in previous sections.

A. Stationary solution

In the limit $t \to \infty$, Eq. (S1) becomes

$$0 = \frac{\partial n(\tau)}{\partial \tau} - n(\tau)J + 2n(0)R(\tau), \quad \text{(S3)}$$

where $J = (\ln 2)/T \int_0^\infty (n(\tau)/K) d\tau$, and $n(\tau)$ does not depend on t. We can solve this equation for the steady-state distribution $n^*(\tau)$:

$$n^*(\tau) = n^*(0) e^{J\tau} \left[1 - 2\int_0^\tau e^{-J\tau'} R(\tau') d\tau'\right], \quad \text{(S4)}$$

with the condition $n^*(\tau \to \infty) = 0$ implying that

$$\int_0^\infty e^{-J\tau} R(\tau) d\tau = 1/2, \quad \text{(S5)}$$

which fixes the value of J. If R is a uniform distribution with mean $\tau = T$ and width $2wT$, we obtain from (S5) that

$$e^{-JT} \frac{\sinh(JTw)}{JTw} = 1/2. \quad \text{(S6)}$$

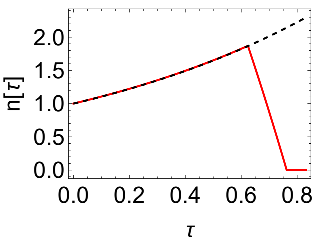

Figure S1. Stationary cell density $n^*(\tau)$ for $T = \ln 2, w = 0.1$. Red line = Eq. (S7). Black dashed line = approximate solution (S8).

This equation must be solved for J numerically. In addition, one must determine the value of $n^*(0)$ from the relationship between J and $n(\tau)$:

$$JK = n^*(0) \int_0^{T(1+w)} e^{J\tau} \left[1 - 2\int_0^{\tau} e^{-J\tau'} R(\tau')d\tau'\right] d\tau \tag{S7}$$

Figure S1 shows an example of $n^*(\tau)$ for $w = 0.1$, calculated in this way. The cell number density is proportional to $e^{J\tau} \approx 2^{\tau/T}$ for $\tau < T$, and rapidly falls down to zero for $\tau > T$. The solution simplifies greatly in the limit $w \to 0$, in which J tends to $(\ln 2)/T$. The steady state number density becomes then

$$n^*(\tau) = K\frac{\ln 2}{T}2^{\tau/T} \tag{S8}$$

B. Evolution of a small perturbation

We now consider the time evolution of a small perturbation to the steady state solution:

$$n(\tau, t) = n^*(\tau)(1 + \epsilon(\tau, t)). \tag{S9}$$

of the noise-less equation S1. Inserting this into Eq. S1 gives:

$$\begin{aligned} n^*\partial_t\epsilon = &(\partial_\tau n^*)(1+\epsilon) + n^*\partial_\tau\epsilon \\ &- \frac{\ln 2}{TK}n^*(1+\epsilon)\int_0^\infty n^*(\tau')(1+\epsilon(\tau',t))d\tau' \\ &+ 2R(\tau)n^*(1+\epsilon). \end{aligned} \tag{S10}$$

We note that $\partial_\tau n^* = Jn^* - 2n^*(0)R(\tau)$, and keep only terms linear in ϵ:

$$\begin{aligned} n^*\partial_t\epsilon = &n^*\partial_\tau\epsilon - \frac{\ln 2}{TK}n^*\int_0^T n^*(\tau)\epsilon(\tau',t)d\tau' \\ &+ 2R(\tau)n^*(0)(\epsilon(0,t) - \epsilon(\tau,t)). \end{aligned} \tag{S11}$$

We divide by n^* and obtain

$$\begin{aligned} \partial_t\epsilon = &\partial_\tau\epsilon - \frac{(\ln 2)}{TK}\int_0^\infty n^*(\tau')\epsilon(\tau',t)d\tau' \\ &- 2\frac{n^*(0)}{n^*(\tau)}R(\tau)(\epsilon(0,t) - \epsilon(\tau,t)) \end{aligned} \tag{S12}$$

with the boundary condition $\epsilon(0,t) = \epsilon(T,t)$. We now expand ϵ and $\frac{n^*(0)}{n^*(\tau)}R(\tau)$ as a Fourier series (consistent with the b.c.):

$$\epsilon(\tau, t) = \sum_{k=-\infty}^{\infty} A_k(t)e^{2\pi ik\tau/T}, \tag{S13}$$

$$\frac{n^*(0)}{n^*(\tau)}R(\tau) = \sum_{k=-\infty}^{\infty} R_k(t)e^{2\pi ik\tau/T}. \tag{S14}$$

where the coefficients $\{R_k\}$ are given by

$$R_k = \frac{1}{T}\int_0^T e^{-2\pi ik\tau/T}\frac{n^*(0)}{n^*(\tau)}R(\tau)d\tau. \tag{S15}$$

The transformed equation reads

$$\begin{aligned} &\sum_k \partial_t A_k e^{2\pi ik\tau/T} = \sum_k A_k(2\pi ik/T)e^{2\pi ik\tau/T} - \\ &-\frac{(\ln 2)}{TK}\sum_k A_k\int_0^T n^*(\tau')e^{2\pi ik\tau'/T}d\tau' \\ &+2\sum_m R_m e^{2\pi im\tau/T}\left(\sum_k A_k - \sum_k A_k e^{2\pi ik\tau/T}\right). \end{aligned} \tag{S16}$$

The sums in (S16) can be compared term by term since they must be valid for any τ. This leads to the following equation for the Fourier coefficients $A_k(t)$:

$$\begin{aligned} \partial_t A_k = &(2\pi ik/T)A_k \\ &- \delta_{k,0}\frac{\ln 2}{TK}\sum_m A_m\int_0^T n^*(\tau')e^{2\pi im\tau'/T}d\tau' \\ &+ 2(R_k\sum_m A_m - \sum_m R_m A_{k-m}). \end{aligned} \tag{S17}$$

In particular, for $k > 0$ we have

$$\partial_t A_k = (2\pi ik/T)A_k + 2(R_k\sum_m A_m - \sum_m R_m A_{k-m}). \tag{S18}$$

Let us assume that the initial perturbation is a pure kth Fourier mode, i.e., $A_k \neq 0$ only for a single value of k. The first term represents oscillations with period T/k of that mode, which essentially gives a travelling-wave type of solution $\sim \exp(2\pi ik(\tau - t)/T)$. The second term represents damping with rate $\gamma_k = -2(R_k - R_0)$. We can calculate this rate using equation (S15) in the limit $w \to 0$, since then we have from Eq. (S8) that $n^*(0)/n^*(\tau) = 2^{-\tau/T}$ and hence

$$R_k = \frac{1}{T}\int_{T(1-w)}^{T(1+w)} e^{-2\pi ik\tau/T}2^{-\tau/t}\frac{1}{2Tw}d\tau. \tag{S19}$$

We obtain that

$$\gamma_k = -2(R_k - R_0) \cong \frac{2\pi^2}{3T}k^2w^2, \tag{S20}$$

which, for $T = \ln 2$ and $k = 1$ reproduces the decay rate $\gamma \approx 9.5w^2$ which we have already seen in Sec. IV A.

C. Amplitude of oscillations for perfectly synchronous replication

We shall now add noise to the model and see how it affects its behaviour. We shall first consider a fully synchronous replication with arbitrary period T, which leads to the following equation:

$$\partial_t n = \partial_\tau n - \frac{\ln 2}{T} n \int_0^T \frac{n(\tau', t)}{K} d\tau' + \sqrt{n^*}\eta, \qquad \text{(S21)}$$

with boundary conditions

$$n(0,t) = (1/2)n(T,t). \qquad \text{(S22)}$$

The noise term $\sqrt{n^*}\eta$ is due to death only, since replication is perfectly synchronous. We assume $\eta(\tau, t)$ represents uncorrelated white noise:

$$\langle \eta(\tau_1, t_1)\eta(\tau_2, t_2)\rangle = D\delta(\tau_1 - \tau_2)\delta(t_1 - t_2), \qquad \text{(S23)}$$

with some $D > 0$ to be specified later. It can be easily verified that this form of noise arises from a master equation for the model with no replication by performing a van Kampen expansion [37] of the master equation. While it may be possible to derive the noise term also in the presence of non-Markovian replication, we find it easier to postulate that Eq. (S23) generally holds, and justify it based on the agreement between the result of our calculation and the computer simulation (see below).

In the absence of noise, equation (S21) has the steady-state solution

$$n^*(\tau) = K\frac{\ln 2}{T} 2^{\tau/T}, \qquad \text{(S24)}$$

$$\int_0^T n^*(\tau) d\tau = K. \qquad \text{(S25)}$$

To solve the time-dependent equation with noise, we consider a small perturbation (similarly as in the previous section):

$$n(\tau, t) = n^*(\tau)(1 + \epsilon(\tau, t)). \qquad \text{(S26)}$$

This gives

$$n^*\partial_t \epsilon = (\partial_\tau n^*)(1+\epsilon) + n^*\partial_\tau \epsilon - \frac{\ln 2}{TK} n^*(1+\epsilon) \int_0^T n^*(\tau')(1+\epsilon(\tau', t))d\tau' + \sqrt{n^*}\eta. \qquad \text{(S27)}$$

We note that $\partial_\tau n^* = ((\ln 2)/T)n^*$, and only keep terms linear in ϵ:

$$n^*\partial_t \epsilon = n^*\partial_\tau \epsilon - \frac{(\ln 2)^2}{T^2} n^* \int_0^T 2^{\tau'/T}\epsilon(\tau', t)d\tau' + \sqrt{n^*}\eta. \qquad \text{(S28)}$$

We divide by n^* and obtain

$$\partial_t \epsilon = \partial_\tau \epsilon - \frac{(\ln 2)^2}{T^2} \int_0^T 2^{\tau'/T}\epsilon(\tau', t)d\tau' + (n^*)^{-1/2}\eta, \qquad \text{(S29)}$$

with the following boundary and initial conditions: $\epsilon(0,t) = \epsilon(T,t)$ and $\epsilon(\tau, 0) = 0$. Proceeding as in the previous section, we expand ϵ and $(n^*)^{-1/2}\eta$ as a Fourier series (consistent with the b.c.):

$$\epsilon(\tau, t) = \sum_{k=-\infty}^{\infty} A_k(t) e^{2\pi k i\tau/T}, \qquad \text{(S30)}$$

$$(n^*(\tau))^{-1/2}\eta(\tau, t) = \sum_{k=-\infty}^{\infty} \eta_k(t) e^{2\pi k i\tau/T}. \qquad \text{(S31)}$$

(S32)

The transformed equation reads

$$\sum_k \partial_t A_k e^{2\pi k i\tau/T} = \sum_k A_k (2\pi i k/T) e^{2\pi k i\tau/T} - \\ -\frac{(\ln 2)^2}{T^2} \sum_k A_k \int_0^T 2^{\tau'/T} e^{2\pi k i\tau'/T} d\tau' \\ + \sum_k \eta_k(t) e^{2\pi k i\tau/T}.$$

The integral over $d\tau'$ gives

$$\int_0^T 2^{\tau'/T} e^{2\pi k i\tau'/T} d\tau' = \frac{iT}{i\ln 2 - 2\pi k}. \qquad \text{(S33)}$$

Comparing the sums in (S33) term-by-term we notice that the $(\ln 2)^2$ term does not contain any factor $e^{2\pi k i\tau/T}$, so it only contributes to the constant term:

$$\delta_{k,0}\frac{(\ln 2)^2}{T}\sum_n A_n \frac{i}{i\ln 2 - 2\pi n} \approx \delta_{k,0}\frac{\ln 2}{T} A_0, \qquad \text{(S34)}$$

where we have assumed that all A_n for $n \neq 0$ are much smaller than A_0 (we shall see later that this is the case). This leads to the following equation for the Fourier coefficients $A_k(t)$:

$$\partial_t A_k = (2\pi i k/T)A_k - ((\ln 2)/T)\delta_{k,0}A_0 + \eta_k. \qquad \text{(S35)}$$

In particular, for $k = 0$ we have

$$\partial_t A_0 = -\frac{\ln 2}{T} A_0 + \eta_0, \qquad \text{(S36)}$$

which can be formally solved as

$$A_0(t) = 2^{-t/T}\int_0^t 2^{t'/T}\eta_0(t')dt'. \qquad \text{(S37)}$$

This gives

$$\left\langle A_0 A_0^\dagger \right\rangle(t) = 2^{-\frac{2t}{T}} \int_0^t dt_1 \int_0^t dt_2 2^{\frac{t_1+t_2}{2}} \left\langle \eta_0(t_1)\eta_0^\dagger(t_2)\right\rangle. \qquad \text{(S38)}$$

Equation (S31) enables us to write

$$\eta_k(t) = \frac{1}{T}\int_0^T \left(\frac{K\ln 2}{T}\right)^{-1/2} 2^{-\frac{\tau}{2T}}\eta(\tau, t)e^{-2\pi i k\tau/T} d\tau. \qquad \text{(S39)}$$

The average of the noise term gives

$$\left\langle \eta_k(t_1)\eta_k^\dagger(t_2)\right\rangle = \frac{1}{TK\ln 2}\times$$
$$\times\int_0^T d\tau_1\int_0^T d\tau_2 2^{-\frac{\tau_1+\tau_2}{2T}}e^{-\frac{2\pi ik(\tau_1-\tau_2)}{T}}\left\langle \eta(\tau_1,t_1)\eta^\dagger(\tau_2,t_2)\right\rangle$$
$$=\frac{D}{K}\frac{\delta(t_1-t_2)}{2(\ln 2)^2}. \tag{S40}$$

We therefore have

$$\left\langle A_0 A_0^\dagger\right\rangle(t) = 2^{-2t/T}\int_0^t \frac{2^{2t'/T}D}{K2(\ln 2)^2}dt'$$
$$=\frac{DT}{K}\frac{1-2^{-2t/T}}{4(\ln 2)^3}. \tag{S41}$$

Proceeding similarly for $k\neq 0$, we obtain:

$$A_k(t) = e^{2\pi ikt/T}\int_0^t e^{-2\pi ikt'/T}\eta_k(t')dt', \tag{S42}$$

from which we obtain that

$$\left\langle A_k A_k^\dagger\right\rangle(t) = \int_0^t dt_1\int_0^t dt_2 e^{-2\pi ik(t_1-t_2)/T}\left\langle \eta_k(t_1)\eta_k^\dagger(t_2)\right\rangle$$
$$=\frac{D}{K}\frac{t}{2(\ln 2)^2}. \tag{S43}$$

We can now calculate the standard deviation of ΔN, the difference between the total number of cells at time t and the average steady-state number:

$$\Delta N(t) = \int_0^T n^*(\tau)\epsilon(\tau,t)d\tau \tag{S44}$$
$$=\int_0^T \frac{K\ln 2}{T}2^{\tau/T}\sum_k A_k(t)e^{2\pi ik\tau/T}d\tau$$
$$=\sum_k A_k(t)\frac{K\ln 2}{T}\int_0^T 2^{\tau/T}e^{2\pi ik\tau/T}d\tau$$
$$=(K\ln 2)\sum_k A_k(t)\frac{i}{i\ln 2-2\pi k}. \tag{S45}$$

This gives (we note that terms $\langle A_k A_n^\dagger\rangle$ with $k\neq n$ vanish):

$$\left\langle|\Delta N(t)|^2\right\rangle = (K\ln 2)^2\sum_{k=-\infty}^{\infty}\frac{\left\langle A_k A_k^\dagger\right\rangle(t)}{(\ln 2)^2+(2\pi k)^2}$$
$$=KD\left[T\frac{1-2^{-2t/T}}{4(\ln 2)^3}+2\sum_{k=1}^{\infty}\frac{t/2}{(\ln 2)^2+(2\pi k)^2}\right]$$
$$=KD\left[T\frac{1-2^{-2t/T}}{4(\ln 2)^3}+t\frac{3(\ln 2)-2}{4(\ln 2)^2}\right]. \tag{S46}$$

In the limit $t\to\infty$ this gives

$$\left\langle|\Delta N(t)|^2\right\rangle \cong KDt\frac{3(\ln 2)-2}{4(\ln 2)^2}\approx 0.0413\,KDt. \tag{S47}$$

Equation (S47) predicts that the amplitude of oscillations for perfectly synchronous replication increases linearly in time. However, recall that our result has been derived under the assumption of a small perturbation. In reality, the amplitude will be limited by non-linear effects.

D. Amplitude of quasi-synchronous oscillations

Let us now consider the case of quasi-synchronous replication. Rather than attempting to solve Eq. (S21) with the extra term $R(\tau)$ as in Eq. (S1), we observe (as argued in subsection S2 B) that the k-th Fourier mode will be damped with rate $k^2\gamma$. We thus consider the following modification to Eq. (S35) for $k\neq 0$:

$$\partial_t A_k = (2\pi ik/T - k^2\gamma)A_k + \eta_k, \tag{S48}$$

where γ is the damping coefficient derived previously. The equation for $k=0$ remains unchanged. Proceeding as in Sec. S2 C, we obtain

$$\left\langle A_k A_k^\dagger\right\rangle(t) = \frac{D}{K}\frac{1-e^{-2k^2\gamma t}}{4(\ln 2)^2 k^2\gamma}. \tag{S49}$$

Inserting this into the equation for $\langle|\Delta N(t)|^2\rangle$ we have in the limit $t\to\infty$:

$$\left\langle|\Delta N|^2\right\rangle = \left\langle|\Delta N(t\to\infty)|^2\right\rangle =$$
$$=KD\left[\frac{T}{4(\ln 2)^3}+2\sum_{k=1}^{\infty}\frac{1}{4k^2\gamma}\frac{1}{(\ln 2)^2+(2\pi k)^2}\right]$$
$$=KD\left[\frac{T}{4(\ln 2)^3}+\pi^2\frac{12-18\ln 2+(\ln 2)^2}{12\gamma(\ln 2)^4}\right]. \tag{S50}$$

For $T=\ln 2$ we obtain that

$$\left\langle|\Delta N|^2\right\rangle \approx KD(0.5203+0.01355/\gamma). \tag{S51}$$

It remains to relate D to the parameters of the model. We again assume that death is the main source of stochasticity, and that the contribution from quasi-synchronous replication is negligible. Consider a pure death process with the same total number of organisms K as the steady state total (S25), and death rate $d=(\ln 2)/T$ as per Eq. (S21). For short time intervals, the variance of the number of organisms in the pure death process equals to $\langle|\Delta N|^2\rangle = (Kd)t = (K(\ln 2)/T)t$ (easy to derive from the general formula on p. 108-109 of Ref. [38]). On the other hand, from Eq. (S46) we have that for small t and $T=\ln 2$,

$$\left\langle|\Delta N|^2\right\rangle \cong KD\frac{3}{4\ln 2}t. \tag{S52}$$

Comparing the two formulas for $\langle|\Delta N|^2\rangle$, we obtain that

$$D=\frac{4\ln 2}{3}\approx 0.924. \tag{S53}$$

Equation (S51) with the above value of D gives Eq. (12) from the main text.

E. Spectrum of fluctuations

We can now obtain a very good analytic approximation for the spectrum of normalized fluctuations $y(t) = N(t)/K - 1$ in the single-species model by Fourier-transforming the expression for ΔN:

$$\tilde{y}(\omega) = (\ln 2) \sum_k \tilde{A}_k(\omega) \frac{i}{i \ln 2 - 2\pi k}, \tag{S54}$$

in which

$$\tilde{A}_k(\omega) = \lim_{L\to\infty} \frac{1}{\sqrt{L}} \int_{-L/2}^{L/2} A_k(t) e^{i\omega t} dt. \tag{S55}$$

We have

$$\left\langle |\tilde{A}_0(\omega)|^2 \right\rangle = \frac{\langle |\tilde{\eta}_0|^2 \rangle}{\left(\frac{\ln 2}{T}\right)^2 + \omega^2}, \tag{S56}$$

$$\left\langle |\tilde{A}_k(\omega)|^2 \right\rangle = \frac{\langle |\tilde{\eta}_k|^2 \rangle}{(\omega T - 2\pi k)^2 + \gamma^2 T^2 k^4}. \tag{S57}$$

in which $\langle |\tilde{\eta}_k|^2 \rangle$ is defined through the Fourier transform like in Eq. (S55), and evaluates to

$$\langle |\tilde{\eta}_0|^2 \rangle = \langle |\tilde{\eta}_k|^2 \rangle = \frac{D}{K 2 (\ln 2)^2} = D_2 K^{-1}, \tag{S58}$$

with $D_2 = \frac{2}{3 \ln 2} \approx 0.962$. This gives

$$K \left\langle |\tilde{y}|^2(\omega) \right\rangle = (\ln 2)^2 \sum_k \frac{\left\langle |\tilde{A}_k|^2 \right\rangle(\omega)}{(\ln 2)^2 + (2\pi k)^2}$$
$$= \frac{D_2}{\left(\frac{\ln 2}{T}\right)^2 + \omega^2} +$$
$$+ \sum_{k=1}^{\infty} \frac{2 D_2 T^2}{(\omega T - 2\pi k)^2 + \gamma^2 T^2 k^4} \frac{(\ln 2)^2}{(\ln 2)^2 + (2\pi k)^2}$$
$$\approx \frac{D_2}{\left(\frac{\ln 2}{T}\right)^2 + \omega^2} +$$
$$+ \frac{2 D_2 T^2 (\ln 2)^2}{[(\omega T - 2\pi)^2 + \gamma^2 T^2][(\ln 2)^2 + (2\pi)^2]} + \dots \tag{S59}$$

where '$\dots$' stand for terms corresponding to higher harmonics which we neglect because they are strongly suppressed by the denominator increasing fast with k. For $T = \ln 2$, we have

$$K \left\langle |\tilde{y}|^2(\omega) \right\rangle \cong \frac{D_2}{1+\omega^2} + \frac{2(\ln 2)^2 D_2}{(\ln 2)^2 + (2\pi)^2} \frac{1}{(\omega - \frac{2\pi}{\ln 2})^2 + \gamma^2}. \tag{S60}$$

The formula as a function of frequency $f = \omega/(2\pi)$ reads:

$$K \left\langle |\tilde{y}|^2(f) \right\rangle \cong$$
$$\cong \frac{D_2/(2\pi)^2}{1 + (2\pi f)^2} + \frac{D_2 \frac{2(\ln 2)^2}{(2\pi)^2}}{(\ln 2)^2 + (2\pi)^2} \frac{1}{(2\pi f - \frac{2\pi}{\ln 2})^2 + \gamma^2},$$
$$\tag{S61}$$

where the factor $1/(2\pi)^2$ is required for correct normalization. Evaluating all numerical factors gives Eq. (13) from the main text.

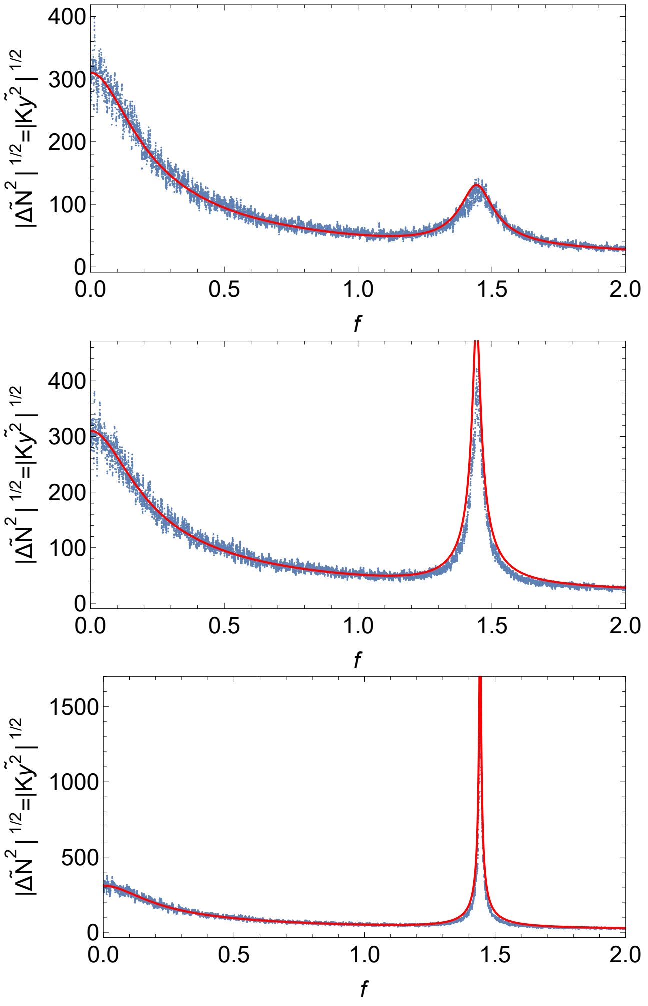

Figure S2. Fourier spectrum $|K\tilde{y}^2|^{1/2}$ of $N(t)$ in the single-species model for $K = 10^5$. Blue points = simulations, red lines = theoretical prediction (no fitting) obtained from Eq. (13). From top to bottom: $w = 0.2, 0.1, 0.05$.

S3. ANALYTICAL DERIVATIONS FOR THE TWO-SPECIES MODEL

A. Spectrum of N_A

We assume that fluctuations around the steady state in the single species model can effectively be described by the following equation:

$$dy/dt = -by + \text{coloured noise}. \tag{S62}$$

Since we know the spectrum of y, we can calculate the spectrum of the coloured noise as $dy/dt + by$ or, in Fourier space, by multiplying Eq. (S60) by $b^2 + \omega^2$:

$$\left\langle |\tilde{\eta}_{A,B}|^2(\omega) \right\rangle = K_{A,B}^{-1} (b^2 + \omega^2) \left(\frac{D_2}{b^2 + \omega^2} + \right.$$
$$\left. + \frac{2(\ln 2)^2 D_2}{(\ln 2)^2 + (2\pi)^2} \frac{1}{(\omega - \frac{2\pi}{T})^2 + \gamma^2} \right), \tag{S63}$$

with $K_A = Kx_A^*, K_B = Kx_B^*$. We then insert Eq. (S63) into Eq. (19), assuming again that $\langle|\eta_{AB}|^2\rangle = 0$ (justified since both species replicate independently with rates unaffected by the other species).

B. Amplitude of oscillations

The variance of $\Delta N_A(t)$ - the difference between the actual $N_A(t)$ and the average number $K_A = Kx_A^*$ of organisms, is given by the following expression:

$$\langle|\Delta N_A(t)|^2\rangle = \frac{K_A^2}{2\pi}\int_{-\infty}^{\infty}\langle|\tilde{y}_A|^2(\omega)\rangle\, d\omega$$
$$= \frac{K_A^2}{2\pi}\int_{-\infty}^{\infty}\Big\{\big[(a_{BB}^2+\omega^2)\langle|\eta_A|^2(\omega)\rangle + a_{AB}^2\langle|\eta_B|^2(\omega)\rangle\big]$$
$$/\left[a_{AB}^2a_{BA}^2 + 2a_{AB}a_{BA}\omega^2 + a_{BB}^2\omega^2 + \omega^4\right]\Big\}d\omega, \qquad \text{(S64)}$$

in which we used Eq. (19) and assumed no correlation between the noise η_A and η_B ($\langle|\eta_A\eta_B|^2\rangle = 0$).

In the case of Poisson replication, we put $\langle|\eta_A|^2\rangle = \langle|\eta_B|^2\rangle = (2b)(K/2)$ and evaluate the integral (S64) numerically. For our usual choice of the parameters $S_{0.5}$, we obtain $\langle|\Delta N_A(t)|^2\rangle_{\text{theor}} \approx 5.48\times10^5$ which is very close to the numerical estimate from the simulation, $\langle|\Delta N_A(t)|^2\rangle_{\text{sim}} \approx 5.56\times10^5$.

In the case of quasi-synchronous replication, we insert Eqs. (S63) into Eq. (S64), and again integrate numerically over ω. This results in Figure 11 in the main text.

C. Transition from Poissonian to synchronous replication

Let us now consider how small w needs to be for the variance to start deviating from the Poisson case, i.e., how synchronous replication must be to differ from random, asynchronous replication. Equation (S64) can be rewritten as follows:

$$\langle|\Delta N_A(t)|^2\rangle = \frac{K_A D_2}{2\pi}\int_{-\infty}^{\infty}F(\omega)(f_0(\omega)+f_\gamma(\omega))d\omega, \qquad \text{(S65)}$$

where

$$F(\omega) = \frac{a_{BB}^2 + a_{AB}^2(K_B/K_A)+\omega^2}{(\omega^2-\Omega^2)^2+c} \qquad \text{(S66)}$$

is the resonance response function, with $c = -a_{AB}a_{BA}a_{BB}^2 - a_{BB}^4/4$, and $\Omega^2 = -a_{AB}a_{BA} - a_{BB}^2/2 = 4\pi^2$ (squared resonant frequency for our parameters $S_{0.5}$). The function

$$f_0(\omega) = \frac{\omega^2}{\omega^2+(\ln 2)^2} \qquad \text{(S67)}$$

is the γ-independent contribution from stochastic replication, and the function

$$f_\gamma(\omega) = \frac{2(\ln 2)^2\omega^2}{(\gamma^2+(\omega-2\pi)^2)(4\pi^2+(\ln 2)^2)} \qquad \text{(S68)}$$

is the γ-dependent contribution.

$F(\omega)$ has full width at maximum height (FWHM) approximately equal to $\sqrt{c}$, whereas $f_\gamma(\omega)$ has FWHM equal to $\approx 2\gamma \approx 13.16w^2$. We expect that when the contribution from $f_\gamma(\omega)$ near the peak of $F(\omega)$ is larger than the contribution from $f_0(\omega)$, the variance of N_A will be dominated by synchronous replication. For $\gamma < \sqrt{c}$, these contributions can be crudely estimated as follows:

$$\int_{2\pi-\sqrt{c}/2}^{2\pi+\sqrt{c}/2} f_0(\omega)d\omega \approx \sqrt{c}, \qquad \text{(S69)}$$

and

$$\int_{2\pi-\gamma}^{2\pi+\gamma} f_\gamma(\omega)d\omega \approx 2/\gamma, \qquad \text{(S70)}$$

so that the contribution from synchronous replication becomes comparable to death-induced noise for $\gamma < 2/\sqrt{c}$, or when $w < 0.55c^{-1/4}$.
